# Alcohol intake and Parkinson's disease risk in the million women study

**DOI:** 10.1002/mds.27933

**Published:** 2019-11-26

**Authors:** Iris Y. Kim, TienYu Owen Yang, Alicia K. Heath, Rachel F. Simpson, Gillian K. Reeves, Jane Green, Sarah Floud, Anna Brown, David J. Hunter, Valerie Beral, Siân Sweetland, on behalf of the Million Women Study Collaborators

**Affiliations:** ^1^ Department of Epidemiology Harvard T.H. Chan School of Public Health Boston Massachusetts USA; ^2^ Nuffield Department of Population Health University of Oxford Oxford United Kingdom; ^3^ School of Public Health Imperial College London London United Kingdom

**Keywords:** alcohol, Million Women Study, Parkinson's disease, prospective studies

## Abstract

**BACKGROUND:**

Alcohol intake may be associated with a lower risk of Parkinson's disease (PD), but findings from previous studies have been inconclusive.

**OBJECTIVE:**

To determine the association between alcohol intake and PD risk in the Million Women Study, a large, prospective study of women in the UK.

**METHODS:**

Between 1996 and 2001, approximately 1.3 million women in the UK, mean age 56 (standard deviation, 5) years, were recruited into the Million Women Study. Information on alcohol intake, lifestyle factors, and medical history was collected at recruitment by questionnaire. Information on incident cases of PD was ascertained by record linkage to national hospital admission records and death registrations. We estimated multivariable‐adjusted relative risks and corresponding 95% confidence intervals using Cox proportional hazards models according to categories of alcohol intake.

**RESULTS:**

During an average of 17.9 years of follow‐up, 11,009 women had a new record of PD among 1,309,267 women. In drinkers, the multivariable‐adjusted relative risk comparing women who drank more than 14 drinks of alcohol per week with women who drank 1 to 2 drinks of alcohol per week was 0.99 (95% confidence interval: 0.90, 1.10). Results did not materially change after excluding the first 10 years of follow‐up (relative risk_adjusted_ = 1.01; 95% confidence interval: 0.90, 1.13). There were no significant trends in alcohol‐related PD risk among never smokers. Additionally, examining this association by type of alcohol intake also yielded null findings.

**CONCLUSION:**

These results do not support an association between alcohol intake and PD risk in women. © 2019 The Authors. *Movement Disorders* published by Wiley Periodicals, Inc. on behalf of International Parkinson and Movement Disorder Society.

Several epidemiological studies have investigated the association between alcohol intake and the risk of Parkinson's disease (PD).^1^, ^2^, ^3^ Results from some studies suggest a modestly lower PD risk^4^, ^5^, ^6^ with increasing alcohol intake whereas others did not find a protective effect.^7^, ^8^, ^9^, ^10^, ^11^, ^12^, ^13^ In addition, investigators of the NIH‐AARP Diet and Health Study reported that low beer consumption was associated with lower PD risk compared to nonbeer drinkers whereas greater liquor consumption was associated with a higher risk, suggesting that the effect of alcohol on PD risk may depend on specific types of alcoholic beverages.^14^

Although the exact mechanism through which alcohol intake may influence PD risk is unknown, there are a few proposed hypotheses. Several other addictive behaviors, such as cigarette smoking and caffeine consumption, may have biological mechanisms that confer neuroprotection against PD.^11^, ^15^, ^16^ It is also possible that individuals who are more vulnerable to developing PD are less likely to smoke or consume caffeine because of damage to the mesolimbic dopaminergic system that makes addictive or novelty‐seeking behaviors unrewarding to them. Under this “premorbid personality” hypothesis,^17^, ^18^, ^19^ alcohol drinking behavior may also be inversely associated with PD risk. Finally, biological components of alcoholic beverages may influence the risk of PD. For example, urate‐elevating effects of beer^20^, ^21^ and flavonoids found in red wine^22^ may be protective against PD.

A meta‐analysis of 32 studies with 9,994 cases among 677,550 participants concluded that alcohol intake, particularly from beer, may be inversely associated with PD risk.^2^ However, a majority of the studies were retrospective studies that were susceptible to recall bias and often had insufficient control for potential confounding factors, and they did not find any significant associations for women. Therefore, we examined the association between alcohol intake and PD risk in a large, prospective cohort of women in the United Kingdom.

## Participants and Methods

### Study Population

Between 1996 and 2001, the Million Women Study recruited approximately 1.3 million women aged 50 to 64 years through National Health Service (NHS) breast screening centers in England and Scotland. At recruitment, participants provided written informed consent and completed questionnaires regarding their lifestyle and medical history (http://www.millionwomenstudy.org). Detailed information on the study design and methods has been described previously,^23^ and information on data access is available through the study website.

All participants were followed for death, incident cancer registration, and emigration by linkage to the NHS Central Registers. Information on NHS inpatient or day‐case hospital admissions was obtained using the Hospital Episode Statistics (HES) in England^24^ from 1 April 1997 and Scottish Morbidity Records from the Information Services Division in Scotland^25^ from 1 January 1981. Causes of death (underlying) and hospital admissions were coded using the International Classification of Diseases, Ninth Revision (ICD‐9) and Tenth revision (ICD‐10).^26^ This study was approved by the Oxford and Anglia Multicentre Research Ethics Committee.

### Assessment of Alcohol Intake

Information on usual alcohol intake was obtained by questionnaire at recruitment. Total alcohol intake was derived from participants’ reported weekly alcohol intake of different types of alcohol (i.e., glasses of wine, half pints of lager/cider/beer, and “tots” [equivalent to “shots”] of spirits), with each alcoholic drink having approximately 10 g of pure alcohol.^27^ There were eight possible multiple‐choice responses for each alcoholic beverage, ranging from “none” to “21+” drinks per week. Repeat measurement of alcohol intake was obtained 14 years later on average by 24‐hour diet recall web questionnaires, which were sent to participants on randomly selected days of the week (n = 44,524). For the current analyses, women were defined as alcohol drinkers if they reported drinking at least one drink per week. Nondrinkers were defined as those reporting drinking 0 or <1 alcohol drink per week.

### Assessment of Other Covariates

Because information on coffee and tea intake and family history of PD (mother or father) was first collected in the 3‐year resurvey, we repeated the analyses considering the 3‐year resurvey as the study baseline.

### Definition of PD Cases

Participants were determined to be incident cases of primary PD if they had an inpatient or day‐case hospital admission record and/or death registration with an ICD‐10 G20 code during the study follow‐up period. Participants with incident secondary parkinsonism (ICD‐10 G21) were censored from the analyses.

### Statistical Analysis

The baseline cohort for this study was comprised of 1,364,308 women who returned questionnaires at recruitment. Women were excluded if: they had a previous hospital admission record of primary PD (ICD‐10 code G20, ICD‐9 code 332.0), had reported having PD on the recruitment questionnaire (n = 1,029), or had a previous hospital record of secondary parkinsonism (ICD‐10 code G21, ICD‐9 code 332.1; n = 6); they had invasive cancer (other than nonmelanoma skin cancer; n = 43,549) before recruitment; they had emigrated or were otherwise lost to NHS follow‐up (n = 83); or if they had missing information on alcohol consumption (n = 10,374). Therefore, the number of eligible participants included 1,309,267 women (Supporting Information Fig. S1).

Participants contributed person‐years starting from the date of recruitment (i.e., study baseline) or 1 April 1997 for women who were recruited in England before HES records began (<5%) until date of first hospital admission at which primary PD or secondary parkinsonism was recorded, date of death, emigration, or the end of the study follow‐up (31 December 2017), whichever occurred first.

We first compared incident PD risk in the following categories of total alcohol intake: nondrinkers, 1 to 2 (ref), >2 to 6, >6 to 14, or >14 drinks per week. Women who reported drinking no alcohol or <1 drink per week at recruitment (n = 468,294) were subsequently excluded from the main analyses given that the large majority are ex‐drinkers (among women who completed a study questionnaire 12 years after baseline, only approximately 1 of 7 women who reported drinking <1 drink per week at baseline were lifelong alcohol abstainers^28^). Their inclusion in the main analyses may have introduced bias if they had stopped drinking because of ill health. In addition, participants who reported being nondrinkers (those who drank 0 or <1 drink per week) at baseline had multiple characteristics (deprivation, education, smoking status, exercise, history of diabetes, hypertension, heart disease, and stroke) that differed considerably from participants who reported drinking (Table 1).

**Table 1:** Characteristics of women at baseline^a^ according to weekly alcohol intake (n = 1,309,267)

	0 to <1 (n = 468,294)	Categories of Alcohol Intake (drinks/week)			
		1 to 2 (n = 250,629)	>2 to 6 (n = 282,179)	>6 to 14 (n = 242,475)	>14 (n = 65,690)
Mean alcohol intake at baseline (drinks/week)	0.2 (0.2)	1.7 (0.4)	4.2 (1.1)	9.7 (2.2)	20.1 (5.0)
g/week	1.6 (2.3)	16.8 (4.4)	41.7 (11.0)	96.7 (22.3)	200.9 (50.4)
Mean alcohol intake 14 years later (g/week)^b^	11.6 (0.6)	32.9 (13.9)	63.2 (20.3)	121.3 (25.5)	208.3 (33.6)
Age, years	57.2 (4.9)	56.7 (4.9)	56.4 (4.8)	56.1 (4.7)	55.8 (4.6)
Body mass index (kg/m^2^)	27.0 (5.2)	26.4 (4.6)	25.9 (4.3)	25.4 (4.0)	25.4 (4.1)
Scotland (%)	8.8	8.3	8.6	8.6	6.9
Most deprived quintile of Deprivation Index (%)	25.3	17.9	15.9	16.0	15.6
No educational qualification (%)	53.6	42.6	36.2	32.6	28.2
Smoking status (%)					
Never	54.8	56.4	53.4	41.7	32.7
Past	22.8	26.6	29.6	35.9	41.2
Current	22.4	17.0	17.1	22.4	26.2
Strenuous exercise at least once per week (%)^c^	30.7	37.6	42.1	43.6	44.0
Diabetes (%)^d^	4.6	2.4	1.7	1.3	1.2
Hypertension (%)^d^	28.0	24.6	22.6	22.2	24.1
Heart disease (%)^d^	6.5	4.5	3.8	3.4	3.1
Stroke (%)^d^	1.7	1.1	0.9	0.9	0.9
Ever use of hormone replacement Therapy (%)	45.2	49.5	52.0	54.7	55.9
Years of follow‐up per woman	17.7 (3.7)	18.1 (3.3)	18.1 (3.2)	18.0 (3.3)	17.8 (3.6)
Number of cases	4,678	2,084	2,153	1,654	440

Values are means(SD) or percentages.

a All characteristics represent those reported from the recruitment questionnaire (1996–2001), unless otherwise indicated.

b Based on 44,524 women who completed the 24‐hour recall online questionnaire 14 years after recruitment.

c “Strenuous” exercise refers to physical activity that is enough to cause sweating or a fast heart rate.

d Hospital admission or self‐reported history of illness or treatment at baseline.

Cox proportional hazard models were used to estimate multivariable‐adjusted relative risks and their corresponding 95% confidence intervals (CIs) using time in the study as the underlying time variable. The analysis was jointly stratified by single years of birth and single years of recruitment to finely control for confounding by age and time. The multivariable regression adjusted for the following covariates: socioeconomic status (quintiles of Townsend deprivation index^29^); region (nine cancer registry regions in England and Scotland); highest educational attainment (no educational qualification, technical, secondary, or tertiary); body mass index (<25, 25.0–29.9, or ≥30 kg/m^2^); smoking status (never, past, current <5, 5–9, 10–14, 15–19, 20–24, or ≥25 cigarettes/day); strenuous physical activity (rarely/never, at least once a week); hormone replacement therapy (HRT) use (ever/never); and hospital admission or self‐reported history/treatment of each of the following cardiovascular conditions: diabetes, hypertension, heart disease, and stroke (yes/no).

**Figure 1:**
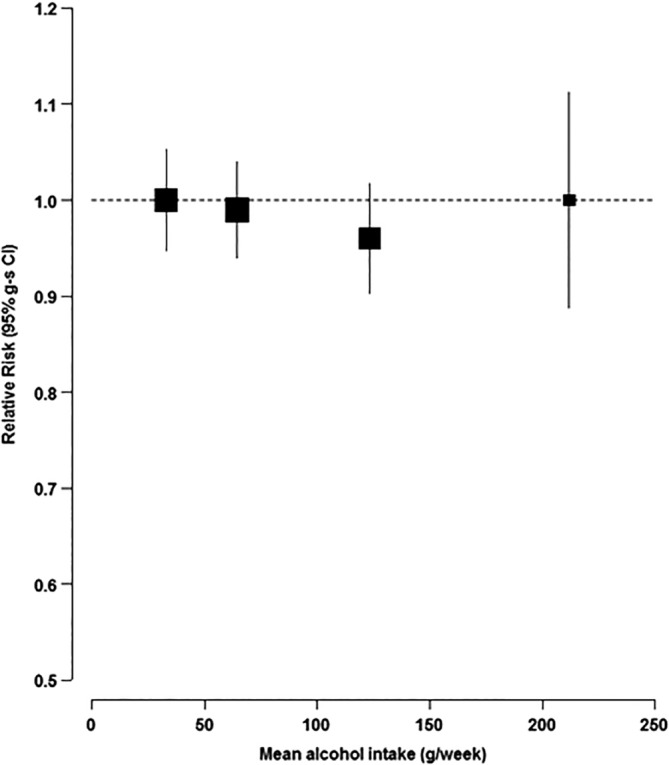
Multivariable‐adjusted RR of PD by alcohol intake among drinkers, excluding the first 10 years of follow‐up. The multivariable‐adjusted RR (95% g‐s CI) for each of the drinking categories (1–2, >26, >6–14, or > 14 drinks per week) are plotted against the repeat measurement of alcohol intake 14 years later for each category (33, 63, 121, and 208 g/week, respectively).

To minimize attenuation of associations by measurement error or changes in alcohol intake over time, we used the regression dilution approach.^30^, ^31^ For this, participants’ alcohol intake was categorized into levels based on their reported alcohol intake at recruitment, and the average intake of alcohol 14 years later was assigned to each baseline category (the alcohol intakes 14 years later were assessed using a web‐based 24‐hour dietary assessment tool among a subset of 44,524 women). For tests of trend, the mean remeasured alcohol intake value within each recruitment category was modeled as a continuous variable. The multivariable‐adjusted relative risks (RRs) of PD are plotted against the mean alcohol values of the web‐based questionnaire in each alcohol category in Figure 1 using group‐specific (g‐s) 95% CI to allow for direct comparison between any two categories.^32^

To minimize possible reverse causation bias, whereby women with preclinical PD change their alcohol intake, further analyses excluded the first 10 years of follow‐up; we also carried out analyses restricted to never smokers, comparing PD risk for different types of alcohol (i.e., wine, beer, and spirits), and excluding women who had cardiovascular disease (CVD)‐related conditions.

Given that some women with PD may not be admitted to the hospital, we assessed the proportion of those with a primary care record of PD, who subsequently had a hospital admission record of PD, using data on a subset of the cohort that had been linked to the UK Clinical Practice Research Datalink (CPRD). In 2017, the CPRD included 8% of the UK population registered with an NHS general practitioner (GP) and routinely recorded diagnoses and prescriptions for GP consultations, as well as information from hospital outpatient clinics, the most likely source of a first diagnosis of PD.^33^

All statistical analyses were conducted using Stata software (version 15.0; StataCorp, 2017; StataCorp LP, College Station, TX). Figures were produced in R (R Core Team, 2017; R Foundation for Statistical Computing, Vienna, Austria).

## Results

Among the 1,309,267 women without a previous record of PD or secondary parkinsonism, there were a total of 11,009 incident primary PD cases observed (108 cases were identified through death records and 10,901 cases were identified through hospital records, among whom 1,127 also had a death record of PD) over a mean follow‐up of 17.9 years (standard deviation: 3.4; Supporting Information Fig. S1). There were 751 participants who were censored at a first hospital record of secondary parkinsonism during follow‐up. The distribution of baseline characteristics across categories of alcohol intake is shown in Table 1. Women with a higher alcohol intake were more likely to smoke and take HRT, but were also more likely to be less deprived, have higher educational attainment, and to participate in strenuous exercise. They were less likely to have a history of diabetes and heart disease.

Among women who were nondrinkers (those who drank 0 or <1 drink per week), there was an increased risk of PD (RR = 1.16; 95% CI: 1.10, 1.22) compared to women who drank 1 to 2 drinks per week (Table 2). However, PD risk was somewhat attenuated across all alcohol categories after ≥10 years of follow‐up compared to the first 10 years of follow‐up. Women who consumed more than 14 drinks per week did not have a significantly different risk of PD compared to women who drank 1 to 2 drinks per week (RR = 0.99; 95% CI: 0.90, 1.10). When risk was modeled linearly in relation to alcohol intake among women who drank at least 1 alcoholic drink per week, the RR per increasing drink per day was 0.98 (95% CI: 0.95, 1.02; p_trend_ = 0.32). This lack of association was evident both in the first 10 years and after ≥10 years of follow‐up, as well as in never smokers only.

**Table 2:** Multivariable‐adjusted RRs (95% CI) of PD by categories of weekly alcohol intake, during entire follow‐up time, the first 10 years of follow‐up only, and excluding the first 10 years of follow‐up

Alcohol (drinks/week)	From Recruitment			First 10 Years of Follow‐up Only*		Excluding First 10 Years of Follow‐up*		Excluding First 10 Years of Follow‐up Among Never Smokers*	
	Cases (n = 11,009)	Age‐Adjusted RR (95% CI)	Multivariable‐Adjusted RR (95% CI)*	Cases (n = 2,133)	RR (95% CI)	Cases (n = 8,876)	RR (95% CI)	Cases (n = 4,937)	RR (95% CI)
0 to <1	4,678	1.18 (1.12, 1.25)	1.16 (1.10, 1.22)^a^	1,016	1.25 (1.10, 1.41)^a^	3,662	1.13 (1.07, 1.20)^a^	2,129	1.14 (1.06, 1.23)^b^
1 to 2	2,084	**1.00 (ref)**	**1.00 (ref)**	392	**1.00 (ref)**	1,692	**1.00 (ref)**	983	**1.00 (ref)**
>2 to 6	2,153	0.96 (0.90, 1.01)	0.97 (0.91, 1.03)	373	0.92 (0.80, 1.06)	1,780	0.98 (0.92, 1.05)	1,021	1.01 (0.93, 1.10)
**>**6 to 14	1,654	0.91 (0.85, 0.97)	0.94 (0.88, 1.01)	277	0.86 (0.74, 1.01)	1,377	0.96 (0.89, 1.03)	653	0.99 (0.90, 1.34)
>14	440	0.94 (0.85, 1.05)	0.99 (0.90, 1.10)	75	0.93 (0.73, 1.20)	365	1.01 (0.90, 1.13)	151	1.13 (0.95, 1.34)
*P* trend among drinkers		*P* = 0.04	*P* = 0.32		P = 0.36		*P* = 0.50		*P* = 0.39

* Additionally adjusted for smoking, region, deprivation index, educational attainment, strenuous exercise, body mass index, diabetes, hypertension, heart disease, stroke, and ever HRT use.

a *P* < 0.0001.

b *P* = 0.001.

Because there was some evidence of reverse causation, whereby women may have altered their drinking behavior because of illness or poor health, we excluded those drinking 0 or <1 drink per week from the main analyses. After exclusion of the first 10 years of follow‐up, there was no difference in RR comparing the highest to lowest category of alcohol intake (RR = 1.01; 95% g‐s CI: 0.88, 1.12; Fig. 1).

Furthermore, we investigated the association between alcohol intake and PD risk separately by type of alcohol (Table 3). We found no association between alcohol intake and PD risk among women who drank only one type of alcohol (i.e., spirits only, beer/lager/cider only, or wine only) or among those who drank more than one type of alcohol (the majority). Baseline characteristics by alcohol type are shown in Supporting Information Table S1.

**Table 3:** Analyses of PD risk according to type of alcohol consumed among drinkers

	Alcohol Type			
	Wine Only (n = 178,632)	Beer/Lager/Cider Only (n = 34,438)	Spirits Only (n = 40,047)	More Than One Type (n = 510,601)
Categories of alcohol intake (drinks/week)	Multivariable‐Adjusted* RR (95% CI)			
1 to 2	1.0 (ref)	1.0 (ref)	1.0 (ref)	1.0 (ref)
>2 to 6	0.95 (0.84, 1.07)	1.23 (0.94, 1.60)	0.93 (0.73, 1.19)	0.99 (0.90, 1.09)
>6	0.91 (0.79, 1.06)	0.95 (0.60, 1.51)	0.96 (0.68, 1.34)	0.99 (0.91, 1.09)

* Adjusted for age, smoking, region, deprivation index, educational attainment, strenuous exercise, body mass index, diabetes, hypertension, heart disease, stroke, and ever HRT use.

These findings were robust in additional analyses, such as excluding women who had CVD‐related conditions (data not shown). Additionally, adjusting for coffee and tea intake and family history of PD did not change results (Supporting Information Table S2).

**Figure 2:**
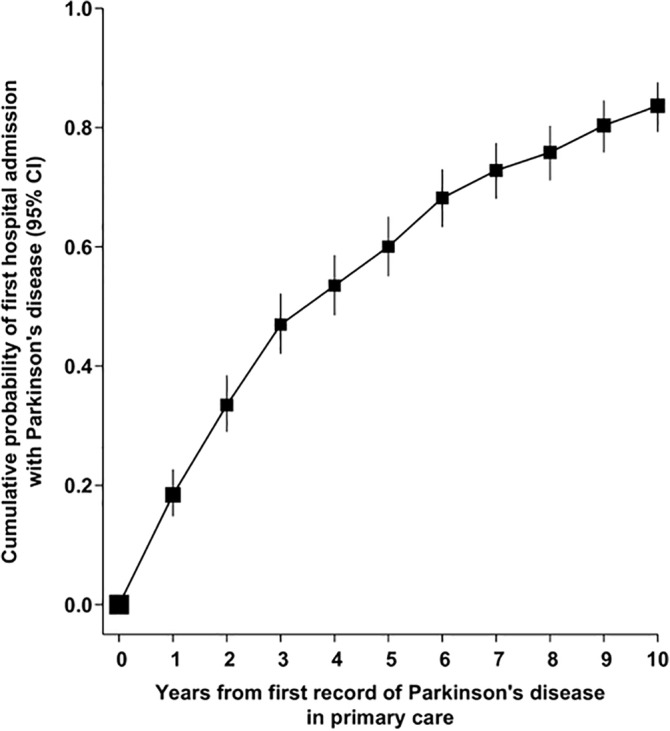
Probability of first admission to hospital with PD, by time since first mention of PD in primary care.

Among the 8% of the cohort for whom primary care data were also available, an estimated 84% of women with a primary care record of PD also had a subsequent hospital admission with an ICD‐10 code for PD during the next 10 years (Fig. 2).

## Discussion

In this analysis of a large, prospective cohort of women in the UK, we found little evidence for an association between usual alcohol intake and PD risk. Nor was there any evidence of an association between intake of specific types of beverages and PD risk.

Some previous studies have suggested a differential effect on PD based on type of alcoholic beverage; drinking beer was associated with a lower incidence of PD in the NHS/HPFS,^12^ whereas drinking spirits was associated with a higher risk in the NIH‐AARP cohort.^14^ In contrast, we did not observe any association with regard to total alcohol intake or type of alcohol. This lack of association is consistent with previous retrospective studies that did not observe an association between alcohol intake and PD risk.^8^, ^9^, ^10^, ^11^ However, it is difficult to interpret these past results given that retrospective studies are more susceptible to recall bias.

Although self‐reported alcohol intake may have been subject to some degree of measurement error in these data, we attempted to address such errors and also changes in alcohol consumption over time by using remeasured values for each baseline category of intake assessed 14 years after baseline. Using this method to allow for regression dilution biases, alcohol intake has been robustly associated with other outcomes, such as cirrhosis^28^ and risk of specific cancers,^27^ in this cohort. As with other observational studies, there may have been other unmeasured or residual confounding. However, we performed several sensitivity analyses, including repeating the analysis among never smokers to minimize potential residual confounding by smoking and repeating the analysis with additional adjustments at the 3‐year resurvey. Other limitations include reduced generalizability to other populations, given that our study was based on UK women. Finally, we could not explore the association of alcohol abstainers because we lacked information to differentiate between lifelong abstainers and past drinkers.

The strengths of this study include its prospective study design, completeness of follow‐up, repeated measures for a subset of women, and large size, with power to detect possible associations. The main limitation is that we used hospital and death registration data to ascertain PD. There is likely to be some delay between the initial diagnosis of PD and the time that it is first recorded in hospital admission data, and participants with hospital admission may represent a subset of cases with more severe disease. However, an estimated 84% of participants with a primary care record of PD go on to have a hospital record of PD within the next 10 years, suggesting that the vast majority of cases will be detected by hospital admission data. In addition, we found that this estimate varied little by alcohol intake at baseline, suggesting that differential ascertainment by alcohol intake is unlikely to have unduly biased the main findings.

In addition, in a recent systematic review that evaluated the accuracy of routinely collected health care data for identifying PD cases, the investigators found that the positive predictive value was >70% for a majority of hospital studies.^34^

To our knowledge, this is the largest prospective study to investigate the association between alcohol intake and PD risk. The results suggest that alcohol intake does not materially influence the risk of PD in UK women.

## Author Roles

(1) Research Project: A. Conception, B. Organization, C. Execution; (2) Statistical Analysis: A. Design, B. Execution, C. Review and Critique; (3) Manuscript Preparation: A. Writing of the First Draft, B. Review and Critique.

Iris Y. Kim: 1A, 1B, 2B, 3A, 3B

TienYu Owen Yang: 1C, 3B

Alicia K. Heath: 1C, 3B

Rachel F. Simpson: 1C, 3B

Gillian K. Reeves: 1C, 3B

Jane Green: 1C, 3B

Sarah Floud: 1C, 3B

Anna Brown: 1C, 3B

David J. Hunter: 1A, 1B, 1C, 3B

Valerie Beral: 1A, 1B, 1C, 3B

Siân Sweetland: 1A, 1B, 1C, 2B, 3B

## Financial Disclosures

Nothing to report.

## Supporting information


**Supplementary table 1** Analyses of PD risk according to type of alcohol consumed
**Supplementary table 2:** Multivariable‐adjusted Relative Risks (95% CI) of Parkinson's disease by categories of total alcohol intake from 3‐year resurvey

**Supplementary Figure 1** Supporting information
