# Supplementary material for: Alcohol intake and Parkinson's disease risk in the million women study
Source: Mov Disord. 2019 Nov 26;35(3):443–9. doi: 10.1002/mds.27933 (PMC7155013; doi:10.1002/mds.27933)
Supplement: Supplementary file 1 — Supplementary table 1 Analyses of PD risk according to type of alcohol consumed Supplementary table 2: Multivariable‐adjusted Relative Risks (95% CI) of Parkinson's disease by categories of total alcohol intake from 3‐year resurvey [file MDS-35-443-s002.docx]

| **Supplementary table 1.** Analyses of PD risk according to type of alcohol consumed | | | | |
| --- | --- | --- | --- | --- |
|  | Alcohol type | | | |
| Characteristics of women at baseline^a,b^ (n=763,718) | Wine-only  (n=178,632) | Beer/lager/cider only  (n=34,438) | Spirits-only  (n=40,047) | More than one type  (n=510,601) |
| Mean alcohol intake (drinks/week) | 5.3 (4.6) | 3.6 (3.9) | 4.4 (3.9) | 7.7 (5.7) |
| g/week | 52.8 (45.8) | 36.0 (38.9) | 43.7 (39.3) | 77.0 (57.1) |
| Mean alcohol intake 14 years later (g/week)^c^ | 87.4 (24.0) | 49.0 (18.3) | 46.7 (15.7) | 111.4 (27.7) |
| Age, years | 56.3 (4.8) | 56.2 (4.8) | 56.6 (4.9) | 56.3 (4.8) |
| Body mass index (kg/m²) | 25.4 (4.1) | 26.4 (4.7) | 26.6 (4.7) | 25.7 (4.2) |
| Scotland | 7.1 | 5.5 | 17.1 | 8.5 |
| Most deprived quintile of Deprivation Index (%) | 10.8 | 28.8 | 22.5 | 17.3 |
| No educational qualification (%) | 25.7 | 53.5 | 49.3 | 37.9 |
| Current smoking status (%) | 10.5 | 26.7 | 26.9 | 20.6 |
| Strenuous exercise at least once per week (%)^d^ | 45.9 | 33.2 | 33.3 | 41.6 |
| Diabetes (%)^e^ | 1.5 | 2.5 | 2.3 | 1.6 |
| Hypertension (%)^e^ | 22.0 | 25.2 | 26.1 | 22.8 |
| Heart disease (%)^e^ | 3.2 | 4.9 | 5.1 | 3.8 |
| Stroke (%)^e^ | 0.8 | 1.3 | 1.3 | 0.9 |
| History of Hormone Replacement Therapy (%) | 51.6 | 49.5 | 51.0 | 53.4 |
| Years of follow-up per woman | 18.2 (3.1) | 17.8 (3.5) | 17.8 (3.5) | 18.0 (3.3) |
| Number of cases | 1,377 | 253 | 330 | 3,688 |
| ^a^ Values are means (SD) or percentages  ^b^ All characteristics represent those reported from the recruitment questionnaire (1996-2001), unless otherwise indicated  ^c^ Based on 33,703 women who completed the 24-hour recall online questionnaire  ^d^ “Strenuous” exercise refers to physical activity that is enough to cause sweating or a fast heart rate  ^e^ Hospital admission or self-reported history of illness or treatment | | | | |

| **Supplementary table 2:** Multivariable-adjusted Relative Risks (95% CI) of Parkinson’s disease by categories of total alcohol intake from 3-year resurvey | | | | | | |
| --- | --- | --- | --- | --- | --- | --- |
|  |  | **Full follow-up** | | | **Excluding first 10 years**  **Model 2** | |
|  |  | **Age-adjusted** | **Model 1** | **Model 2** |  |  |
| **Alcohol (drinks/week)** | **Cases**  **(n=2,507)** | **RR (95% CI)** | **RR (95% CI)** | **RR (95% CI)** | **Cases**  **(n=1,714)** | **RR (95% CI)** |
| 1-2 | 577 | **1.00 (ref)** | **1.00 (ref)** | **1.00 (ref)** | 385 | **1.00 (ref)** |
| >2-6 | 899 | 1.04 (0.93, 1.15) | 1.05 (0.95, 1.17) | 1.05 (0.95, 1.17) | 608 | 1.06 (0.93, 1.21) |
| **>**6-14 | 811 | 0.98 (0.88, 1.09) | 0.98 (0.88, 1.10) | 0.99 (0.88, 1.10) | 559 | 0.99 (0.86, 1.13) |
| >14 | 220 | 0.98 (0.84, 1.15) | 1.01 (0.86, 1.19) | 1.02 (0.87, 1.19) | 162 | 1.06 (0.88, 1.29) |
| Model 1: Adjusted for age, smoking, region, deprivation index, educational attainment, strenuous exercise, body mass index, self-reported history of diabetes, hypertension, heart disease, stroke, and ever HRT use  Model 2: Additionally adjusted for family history of PD (mother or father), coffee and tea consumption | | | | | | |
