# Supplementary figures and images for: Alcohol intake and Parkinson's disease risk in the million women study
Source: Mov Disord. 2019 Nov 26;35(3):443–9. doi: 10.1002/mds.27933 (PMC7155013; doi:10.1002/mds.27933)

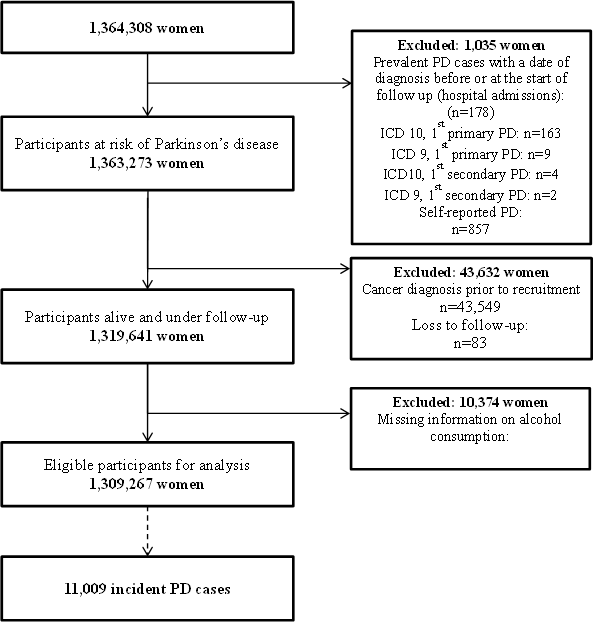

Supplement: Supplementary file 2 — Supplementary Figure 1 Supporting information [file MDS-35-443-s001.tif]
